# Editorial: Critical role of spectroscopic analysis for the development of π-conjugated materials for optoelectronics

**DOI:** 10.3389/fchem.2023.1182156

**Published:** 2023-03-30

**Authors:** Ji Ma, Daohai Zhang, Haichang Zhang, Maning Liu

**Affiliations:** ^1^ Key Laboratory of Rubber-Plastics of Ministry of Education/Shandong Province (QUST), School of Polymer Science and Engineering, Qingdao University of Science and Technology, Qingdao, China; ^2^ School of Chemical Engineering of Guizhou Minzu University, Guiyang, China; ^3^ Centre for Analysis and Synthesis, Department of Chemistry, Lund University, Lund, Sweden

**Keywords:** organic light-emitting diodes, flame retardant, optoelectronics, molecular structure, π-conjugated materials

Over the last decade, researchers have made extensive efforts to develop novel π-conjugated materials for their various applications such as organic-field effect transistors (OFETs), organic solar cells (OSCs), hole-transport materials (HTMs) particularly for halide perovskite solar cells (HPSCs), organic light-emitting diodes (OLEDs), flame retardants, etc. (Meti et al., 2020) To obtain high-performing π-conjugated materials, the rational design of molecular structure is crucial. In this Research Topic, eight original works or minireviews have been reported, including a series of studies on the applications of new π-conjugated materials-based flame retardants and OLEDs. These studies have comprehensively explored different π–conjugated systems from various points of view with a highlight of the spectroscopic analysis.

During almost the last one century, general plastics and engineering plastics have been progressively developed, which play a significant role in influencing and optimizing people’s modern life. However, since the major components of plastics are traditional polymers, whose conjugated backbones are constructed mainly based on the carbon elements, thus, it is quite easy to ignite this kind of plastic products compared with other ceramic or metal-based materials. To this end, many researchers have extensively investigated the flame retardancy of plastics by developing new types of π–conjugated materials. In (Sun et al.)’s work, a conjugated flame-retardant called P-PPD-Ph was successfully synthesized, which was mixed with Poly (lactic acid) (PLA) matrix for forming PLA/P-PPD-Ph. As a new conjugated flame-retardant, the as-synthesized PLA/P-PPD-Ph features low storage modulus/loss modulus as well as complex viscosity parameters, which can promote the formation of high-quality carbon layer. One promising advantage of this sort of flame-retardant is that it can release the phosphorus-containing free radicals during the decomposition of P-PPD-Ph part, which further capture the H·, O·, or HO· radicals by retarding the flame during combustion. Moreover, the addition of P-PPD-Ph in the conjugated system can produce more CO thus less CO_2_, leading to incomplete combustion. Continuously, (Tan et al.) reported a modified conjugated structure of PLA/P-PPD-Ph/epoxy chain extender (ECE) as a new type of flame-retardant, by together adding ECE and P-PPD-Ph during the PP extrusion process. The authors found that both the amount of residual carbon and the limiting oxygen index (LOI) of the composites increase with the increase in the ECE content, which makes a positive effect on retarding the flame. Interestingly, compared with the previous work (Sun et al.), more phosphorus-containing radicals can be generated in the PLA chain with the help of P-PPD-Ph, which not only enhances the cohesive phase of flame-retardant, but also improves the bending, tensile and impact strengths.

It is known that Polyamide 6 (PA6), poly (ethylene terephthalate) (PBT) and polypropylene (PP) similarly belong to one type of attractive engineering plastics. On the other hand, the common halogen-free flame retardants such as metal phosphates usually exhibit bad mechanical properties. To solve this Research Topic, (Gao et al.), simultaneously improved the flame retardancy and mechanical properties of PA6 by employing two types of 9,10-dihydro-9-oxa-10-phosphaphenanthrene-10-oxide (DOPO)-based conjugated flame retardants, namely PA6/DIDOPO and PA6/DOPO-NH_2_. PA6/DOPO-NH_2_ shows better flame retardancy compared to that of PA6/DIDOPO. Their differential scanning calorimetry (DSC) and rheological analysis demonstrate that the strong interaction between DOPO-NH_2_ and PA6 is the key to improve the mechanical properties. The authors also claimed that the amino-terminal group can interact with the carboxyl group within PA6, which is responsible for the improvement of the compatibility between flame retardant and PA6 matrix. This work provides an alternative and promising strategy for design and synthesis of more functional flame retardants. Moreover, PBT is also known as a highly flammable material with a LOI of 20%–22%, which can generate a lot of heat during combustion, resulting in serious melt dripping. (Sun et al.) investigated the influence of conjugated molecule 10-(2,5dihydroxy phenyl)-10H-9-oxo-10-phosphofi-10-oxide (DOPO-HQ) on the flame-retardant properties of PBT. They found that DOPO-HQ, occupying 14% of the total PBT structural space, can exert phosphorus oxygen radicals (PO·) in gas phase during combustion, which can terminate the chain radicals by trapping hydrogen radicals (H·) and hydroxyl radicals (HO·), aiming at an incomplete combustion in gas phase. Later on, (Zhang et al.) studied the flame-retardant effect of 9,10-dihydro-9-oxa-10-phosphaphenanthrene-10-oxide derivative (DiDOPO) in a conjugated structure with PP. The authors discovered that by adding 16 wt% of DiDOPO, the LOI value of PP-based composite increases from 18.8% (without DiDOPO) to 24% (with DiDOPO), eventually achieving a V-0 fire rating. In addition, the loss modulus of PP/DiDOPO conjugated composite is lower than its storage modulus in the whole frequency range, indicating the occurrence of the so-called “solid-to-liquid” transformation within the material.

Besides, there have been other two reports to explore the potential of conjugated materials in the application of organic electronics. In (Zhan et al.)’s work, the band gap of lateral heterojunctions (LHS) was tuned by tailoring the length (measured by the number of Sb or Bi atomic chains) of the molecular structure. They found that this kind of Sb/Bi-based LHS can absorb light in the near-infrared (NIR) range. Their work demonstrates that the elements in Group VA are promising as the candidates for the construction of LHS in terms of future application of NIR optoelectronics. Moreover, OLEDs have recently attracted more attention in the fields of solid state lighting and display due to their self-illumination, high flexibility, high color purity and low energy consumption. (AlSalhi et al., 2011) (Li et al.) briefly summarized the recent progress of inverted singlet–triplet (INVEST) molecules in the application of OLEDs from both theoretical and experimental study points of view. For an INVEST molecule that possesses a negative singlet-triplet energy gap (ΔEST), the down-converted reverse intersystem crossing (RISC) does not require a thermal activation, which provides a hint to solve the problems of low roll-off efficiency and short lifetime of OLEDs. Benefiting from this feature, INVEST molecules have been recently considered as the next-generation of high-performance organic light-emitting materials. In addition, compared with green or red exciplexes, blue exciplexes are relatively lack of investigation owing to their largely red-shifted spectra and broadened emission range. (Li et al.) provided an overview of the recent progress of blue exciplexes in the application of blue phosphorescent OLEDs. This review sheds light on the future development of high-performance blue exciplex-based OLEDs.

In summary, among the eight articles published in this Research Topic, five papers investigated the positive influence of a series of new organic π-conjugated molecules on the flame retardation for conventional polymer materials, while other three reports emphasized the significant role of organic conjugated molecules in the fabrication of high-performance OLEDs. These articles pave the way for novel π-conjugated functional materials for their future wide applications.

## References

[B1] AlSalhi M. S. Alam J. Dass L. A. Raja M. (2011). Recent advances in conjugated polymers for light emitting devices. Int. J. Mol. Sci. 12, 2036–2054. 10.3390/IJMS12032036 21673938 PMC3111649

[B2] Meti P. Park H. H. Gong Y. D. (2020). Recent developments in pyrazine functionalized π-conjugated materials for optoelectronic applications. J. Mater. Chem. C 8, 352–379. 10.1039/C9TC05014K

